# Effects of effective stereotactic radiosurgery for brain metastases on the adjacent brain parenchyma

**DOI:** 10.1038/s41416-020-0853-3

**Published:** 2020-05-04

**Authors:** Sabine Wagner, Heinrich Lanfermann, Walter Alexander Wohlgemuth, Hubert Gufler

**Affiliations:** 1 Institute of Neuroradiology, Johann-Wolfgang-Goethe-University Frankfurt, Frankfurt/Main, Germany; 2 Department of Neuroradiology, Friedrich-Schiller-University Jena, Jena, Germany; 3 Institute of Diagnostic and Interventional Neuroradiology, Hannover Medical School, Hannover, Germany; 4 Clinic and Policlinic of Radiology, Martin-Luther-University Halle-Wittenberg, Halle/Saale, Germany

**Keywords:** Surgical oncology, Cancer imaging, Metastasis, Radiotherapy, CNS cancer

## Abstract

**Background:**

To evaluate whether functional and metabolic MRI can detect radiation-induced alterations in the adjacent areas after effective stereotactic radiosurgery (SRS) for brain metastases. If confirmed, these techniques may be suited for monitoring the timely stratification of patients for neuroprotective treatments after irradiation.

**Methods:**

Inclusion criteria were complete response, partial response, or stable disease on routine follow-up MR-scans. Multiparametric 3T-MRI was performed with diffusion-weighted imaging, dynamic susceptibility perfusion-weighted imaging, and two-dimensional proton MR-spectroscopy. Parameters were measured in the SRS-treated target and in the adjacent parenchyma up to both 0.75 cm and 1.5 cm from the target border.

**Results:**

Nineteen lesions in sixteen consecutive patients met the inclusion criteria. The median follow-up time was 39 months (range, 10–142) with 41 multiparametric MR-examinations in total. We found low values of N-acetyl-aspartate up to 1.5 cm from the target borders of SRS (*P* = 0.043) associated with high values of choline (*P* = 0.004) at the end of the observation period. Lactate levels in the adjacent tissue declined over time, whereas continuously high apparent-diffusion-coefficient values were noted (*P* < 0.001).

**Conclusion:**

Multiparametric MRI can depict radiobiological effects and their time course at a distance from the effectively treated site after SRS for brain metastases, even if conventional MRI findings are inconspicuous.

## Background

Brain metastases are the most common brain tumours, seen in about 20–40% of all adult patients with systemic malignancies.^1,2^ Stereotactic radiosurgery (SRS) is an established treatment option for patients with up to three brain metastases, good performance status and controlled extracranial disease.^1,2^ SRS delivers focused, high-dose, single-fraction irradiation to a defined small target volume. Although the surrounding unaffected healthy brain parenchyma is spared by a steep dose gradient, various patterns of radiation-induced CNS injury have been reported.^3,4^ Different hypotheses have been advanced for the underlying pathophysiology, but the pathological mechanism still remains poorly defined and the possibilities to quantify the extent of injury are limited.^5,6^

Optimal use of SRS as a treatment technique requires an understanding of the effects on both the target volume and the surrounding normal brain tissue.^7^ In an attempt to investigate the cellular and molecular responses of normal brain parenchyma to radiosurgery, several working groups have used animal models, irradiating healthy brain parenchyma in the absence of any pathological substrate, such as a tumour.^7,8^ Tumours and the brain parenchyma, however, interact through an individual microenvironment consisting of complex multiple-cell systems, where microglia and macrophages secrete cytokines, growth factors, enzymes and reactive oxygen species, which in turn trigger angiogenesis, tumour proliferation, and invasion of metastatic cells.^9^ Each tumour entity treated with radiosurgery poses a different risk to the surrounding brain parenchyma of developing radiation injury.^3,10^

In a daily clinical setting, there is no need to apply advanced MRI methods nor is there any justification to histologically verify a response to treatment based on follow-up MRIs. Therefore, information on radiobiological responses in healthy tissue adjacent to metastases effectively treated with SRS is lacking. This study aims to answer the question of whether functional and metabolic MRI can depict radiobiological responses in the healthy tissue adjacent to metastases effectively treated with SRS and, secondly, if this is confirmed, whether the extent of injury to the healthy tissue is time dependent or if there is even brain repair over time.

## Materials and methods

### Patients and treatment

Enrolment was restricted to patients previously treated with SRS for cerebral metastases with complete response (CR), partial response (PR) or stable disease (SD). Response to treatment was assessed radiologically at routine follow-up 1.5-T MRI examinations carried out every 3–6 months after SRS treatment. The study was approved by the local ethics committee. All patients gave their informed consent.

Response to treatment was classified according to the response criteria for CNS metastases proposed by the Response Assessment in Neuro-Oncology (RANO) group for brain metastases (BM).^11^ For SRS, a gamma knife was used with multiple isocentres for a highly conformal dose distribution. The prescription dose was determined by coverage of approximately 95% of the target volume as defined on the stereotactic MRI. Dosing for SRS was based on lesion volume: 20–25 Gy for small to medium brain metastases, and lower for large tumours to avoid complications. Some of the participants of this study had been part of an earlier analysis dealing with different clinical aspects. Exclusion criteria were contraindications for 3-Tesla MRI and for gadolinium-diethylenetriamine pentaacetic acid (Gd-DTPA) contrast medium administration.

### MRI data acquisition

MRI was performed on a 3-Tesla system (Magnetom Allegra, Siemens Healtheneers, Forchheim, Germany) with a quadrature 4-channel head coil. The protocol included standard clinical echoplanar diffusion-weighted MRI (DWI), dynamic susceptibility-weighted contrast-enhanced (DSC) MRI, a 3D high-resolution T1-weighted (T1w) gradient-echo sequence (GRE), T1w spin echo (SE), and proton MR spectroscopic imaging (^1^H-MRSI) (Table 1). DWI was performed using a single-shot, spin echo-type echoplanar sequence (echo time 66.6 ms, repetition time 1.100 ms, field of view 240 × 240 mm, slice thickness 5 mm, matrix 128 × 128 pixels, b = 0 mm^2^/s, and b = 1.000 mm^2^/s). Data with b = 0 provided a set of T2-weighted (T2w) images. DSC-MRI was acquired with an echo time of 33 ms and repetition time of 1.530 ms, field of view 240 × 240 mm, slice thickness 5 mm, and 128 × 128 pixels, 18 slices with 50 repeated images for each slice over 50 s. DSC-MRI was performed after standardised intravenous contrast agent injection (0.1 mmol/kg Gd-DTPA) with a flow rate of 5 ml/s followed by a 30 ml bolus of 0.9% saline. After DSC-MRI, a 3D high-resolution T1w GRE was acquired (echo time 4.38 ms, repetition time 2.500 ms, matrix size 256 × 192, section thickness 1 mm, field of view 240 × 240 mm). The ^1^1H-MRSI was positioned on the image with the largest cross-sectional diameter of the lesion. A point-resolved spectroscopy pulse sequence (PRESS) was used (echo time 144 ms, repetition time 1.500 ms, 2 acquisitions) with a weighted, circular, phase-encoding scheme applied on a 28 × 28 matrix, which was extrapolated to 32 × 32, field of view 240 × 240 mm, 10 mm slice thickness, and voxel size 7.5 × 7.5 × 10 mm.

**Table 1:** Overview of the advanced MR techniques used. Full explanation of the abbreviations and brief description of the technology, naming the most important parameters or the most important metabolites in ^1^H-MRS and their clinical significance.

Functional MRI
*DWI*
Diffusion-weighted imaging
Assessment of tissue microstructure and corresponding pathological processes by characterising the microscopic diffusion of free water molecules, the magnitude of which is quantified as ADC (apparent-diffusion coefficient).^35^
↓ ADC cytotoxic oedema or increased cellularity (e.g. tumour)
↑ ADC vasogenic oedema or decreased cellularity (e.g. necrotic tissues)
*PWI*
Perfusion-weighted imaging
Assessment of haemodynamic information about relative blood volume (rCBV) and flow (rCBF), characterising the microvascular environment in the tissue. In DSC (dynamic susceptibility-weighted contrast-enhanced) perfusion, after a single bolus of contrast medium, images are acquired tracking the temporal course of contrast material passing through the cerebral circulation.
↓ rCBV | rCBF e.g. radiation injury
↑ rCBV | rCBF e.g. tumour
*Metabolic MRI*
^1^H-MRSI
Proton magnetic resonance spectroscopic imaging
MRS is a noninvasive analytical technique for detection and quantification of selected metabolites in vivo. Based on the same physical principals as MRI, MRSI creates a two-dimensional map of spectra. It provides a tool for studying the metabolic profile of pathological processes.
NAA N-acetyl-aspartate marker for neuronal health, viability, and cell number
Cho choline cell membrane precursors and breakdown products
Cr creatine storage and transport of cellular energy
Lac lactate carboxidative metabolism, anaerobic glycolysis

### Data processing and MRI data analysis

**Fig. 1:**
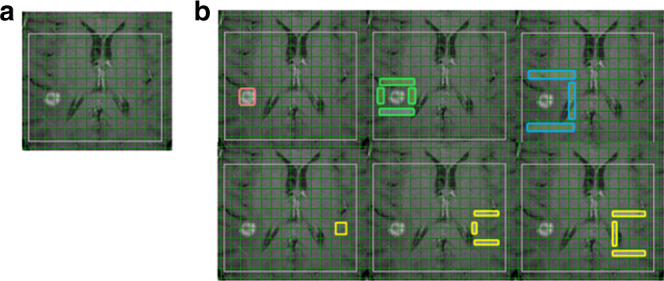
^1^H-MRSI measurements. **a** T1-weighted reference slice depicting the contrast-enhancing metastasis treated by stereotactic radiosurgery (SRS). The grid shows the extrapolated 2D ^1^H-MRSI matrix with the white margin indicating the volume of interest (VOI). **b** For spectra classification the voxels were categorised into (top row) (i) core—representing the enhancing lesion in the former target of SRS (red), (ii) 1st ring—indicating the immediately adjacent voxels in the surrounding brain parenchyma (green), (iii) 2nd ring—given by the next row of voxels (blue), and (lower row) (iv) the corresponding contralateral normal white matter of each ring (yellow).

The MR spectral raw data of ^1^H-MRSI were analysed by using the commercially available package LCModel software.^12,13^ For image-guided selection of ^1^H-MRSI voxels, the grid of ^1^H-MRSI was laid over the contrast-enhanced T1w images: the voxels containing contrast-enhancing tissue of the lesion were defined as core, the immediately adjacent voxels around the core formed the 1st ring, and the next row of voxels gave the 2nd ring, together forming three concentric rings (Fig. 1). The highest value of the trimethylamine groups of phosphocholine, glycerophosphocholine, and free choline (Cho) as well as of creatine and phosphocreatine (Cr) and lactate (Lac) and the lowest value of N-acetyl-aspartate and N-acetyl-aspertylglutamate (NAA) were determined for each ring (consensually by two experienced radiologists [S.W., H.G.]). Normalisation of each metabolite was obtained by calculating the quotient of the value in the lesion and the value in the corresponding voxel in the contralateral hemisphere.

**Fig. 2:**
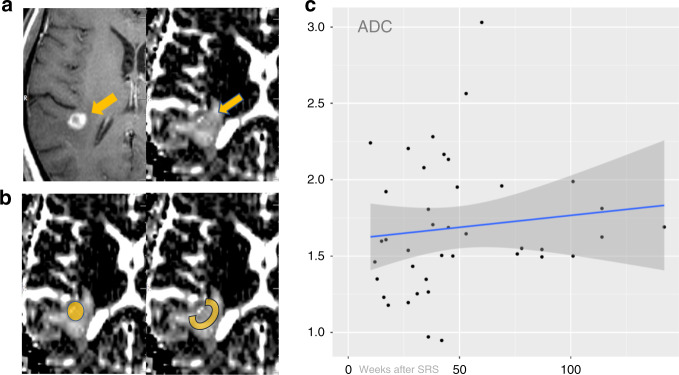
Apparent-diffusion coefficient (ADC) analysis. **a** Contrast-enhanced T1-weighted image (left) and corresponding ADC map (right) obtained in a patient with a metastasis from renal cell carcinoma (arrows) within the right temporal lobe 17 weeks after stereotactic radiosurgery (SRS). **b** Region of interest placement in the contrast-enhancing lesion (left) and in the parenchyma adjacent to the target borders of SRS (right). **c** Scatter plot with fitted regression function for ADC in the parenchyma adjacent to the target borders of SRS. Solid circles: normalised values for each lesion. Solid bold lines: fitted regression line; darkened areas: 95% confidence interval. Note: continuously elevated ADC values throughout the observation period.

Maps of apparent-diffusion coefficients (ADC) were provided automatically by the MR scanner software. ADC values were expressed in 10^−3^ mm^2^/s. Perfusion data were analysed with the Syngo Software (Siemens Healthineers, Forchheim, Germany). Grey-coded regional cerebral blood volume (rCBV) maps and regional blood flow (rCBF) maps were generated. The maps were co-registered with the axial contrast T1w images or with the grid of ^1^H-MRSI, and then displayed as overlays. For quantitative analysis, a polygonal region of interest (ROI) was drawn (consensually by S.W. and H.G) covering (i) the entire contrast-enhancing lesion (for ADC and PWI), (ii) the contrast-enhancing rim (PWI), and (iii) the surrounding brain parenchyma adjacent to the lesion (for ADC and PWI), the latter being congruous with the 1st ring of the grid (Fig. 2a, b). For normalisation a similar small ROI was placed in mirrored white matter regions.

If the enhancing disease had completely disappeared, the residual T2w lesion or the anatomical location in the initial investigations was set as core of the three rings to evaluate ^1^H-MRSI and to assess the diffusion and perfusion maps.

The mirrored contralateral voxels in the healthy, non-irradiated brain served as a reference for determining whether pathology was present in the SRS-treated target and in the adjacent parenchyma. Criteria for the existence of a radiation-induced injury were: elevated ADC values representing a reduced cellularity compared to the healthy opposite side; reduced NAA values (reduction in healthy neurons); elevated Cho values (increased cell metabolism of cell membrane precursors and breakdown products); elevated Cr values (increased energy requirement), and a reduction in CBF and CBV compared to the healthy opposite side. In addition, the positive detection of Lac was attributed to radiation-induced injury.

### Statistical analysis

For statistical analysis we used R Statistics software.^14^ The quantitative measurements and the temporal changes of the metabolic and functional parameters were compared using the paired Student *t*-test. Family of *P*-values was adjusted for multiple testing using Holm’s method. Dependencies between parameters were analysed by pairwise Pearson correlations. For all statistical tests, the significance level was set at *p* < 0.05.

## Results

According to our inclusion criteria, sixteen consecutive patients were prospectively enrolled in this study. The median age at SRS treatment was 52 years (range, 34–68 years). The male:female ratio was 10:6. Primary tumour type/ site was malignant melanoma (four), lung (three), breast (three), renal cell carcinoma (two), colorectal carcinoma (two), synovial sarcoma (one) and testicular carcinoma (one). The number of brain metastases at SRS treatment was one in 14 patients, two in one patient, and three in one patient, respectively.

As a result, sixteen patients with a total of nineteen brain metastases were eligible for the present study. The median follow-up time was 39 months (range, 10–142 months), with a total of 125 routine follow-up scans and 41 multiparametric MR examinations according to the study protocol. ^1^H-MRSI data could not be evaluated in six examinations due to the low quality of spectra caused by motion artefacts, and the PWI data were missing in one MR measurement.

During the follow-up period the size of the enhancing lesion and the T2w signal in the surrounding volume decreased (*P* < 0.01). Complete disappearance of the disease without any residual signal changes on morphological MRI was observed in four patients at four multiparametric MR examinations.

**Fig. 3:**
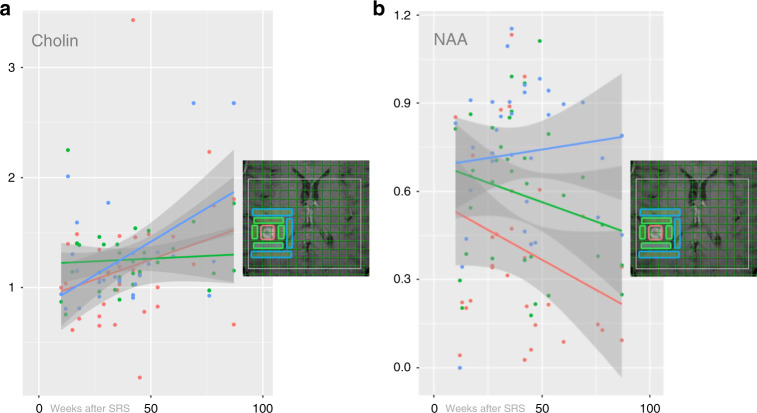
Scatter plot with fitted regression function for metabolites. Choline (Cho) (**a**) and N-acetyl-aspartate (NAA) (**b**): the solid coloured circles represent the normalised values of the core (red), the 1st ring (green), and the 2nd ring (blue). The solid bold lines display the fitted regression line for each variable, with darkened areas representing the 95% confidence interval. Inlays: T1-weighted reference slice is given with the overlaid grid of the ^1^H-MRSI and the color-coded localisation of the measurements in each case.

Normal values were found throughout the observation period for Cr of the 1st and 2nd ring, and for the perfusion parameters of the adjacent tissue (Table 2). Comparing the normalised values in the time period up to 27 weeks with those 52 weeks after SRS, a significant increase in Cho of the 2nd ring (*P* = 0.028) (Fig. 3) and in rCBV of both the entire lesion (*P* = 0.036) and the contrast-enhancing rim (*P* = 0.044) was observed. In contrast, Lac in the core decreased in the corresponding time periods (*P* = 0.049).

**Table 2:** Mean and SD of metabolites, ADC, rCBV and rCBF of the diseased and the contralateral healthy side <27 weeks and >52 weeks after stereotactic radiosurgery (SRS).

Variable		<27 weeks	Diseased *n* = 11 Mean ± SD	Contralateral Mean ± SD	*P-*value adjusted	**>**52 weeks	Diseased *n* = 11 Mean ± SD	Contralateral Mean ± SD	*P-*value adjusted
^*1*^*H-MRSI*									
*Cho*									
Center			1.43 ± 0.61	1.32 ± 0.42	1		1.24 ± 0.38	1.00 ± 0.21	0.72
1st ring			1.78 ± 1.00	1.38 ± 0.51	0.948		1.31 ± 0.34	1.02 ± 0.35	**<0.001**
2nd ring			1.73 ± 0.95	1.54 ± 0.53	1		1.33 ± 0.36	0.96 ± 0.43	**0.004**
*NAA*									
Center			2.98 ± 1.70	7.03 ± 2.81	**0.015**		1.63 ± 1.28	5.43 ± 1.88	**0.002**
1st ring			3.74 ± 1.40	7.02 ± 3.26	0.106		2.87 ± 1.80	5.26 ± 1.33	**0.01**
2nd ring			5.18 ± 1.77	6.48 ± 4.76	0.858		3.78 ± 1.50	5.01 ± 1.67	**0.043**
*Cr*									
Center			3.72 ± 1.42	5.51 ± 1.73	0.19		3.29 ± 1.57	3.88 ± 0.88	1
1st ring			5.07 ± 1.09	5.40 ± 1.34	1		4.06 ± 1.28	4.12 ± 1.61	1
2nd ring			5.90 ± 2.18	5.25 ± 1.63	1		4.55 ± 1.38	4.32 ± 1.64	1
*Lac*									
Center			3.37 ± 2.70	0.27 ± 0.35	**0.026**		1.52 ± 1.75	0.45 ± 0.59	0.193
1st ring			2.34 ± 2.37	0.20 ± 0.23	0.115		1.24 ± 0.38	1.00 ± 0.21	**0.013**
2nd ring			1.30 ± 2.15	0.18 ± 0.26	0.72		1.31 ± 0.34	1.02 ± 0.35	0.11
*DWI*									
*ADC*									
lesion			122.50 ± 24.62	76.10 ± 8.27	**<0.001**		129.04 ± 33.02	72.39 ± 7.49	**<0.001**
adjacent			126.39 ± 28.59	75.80 ± 7.36	**<0.001**		137.67 ± 51.69	72.52 ± 13.82	**<0.001**
*PWI*									
*CBV*									
lesion			338.88 ± 174.85	197.11 ± 159.99	**<0.001**		455.01 ± 203.77	150.00 ± 151.00	**0.003**
c-e rim			523.11 ± 393.38	197.11 ± 159.99	**<0.001**		589.84 ± 167.32	150.00 ± 151.00	**<0.001**
adjacent			226.33 ± 159.34	300.55 ± 209.56	1		191.32 ± 169.09	219.09 ± 151.02	1
*CBF*									
Lesion			53.00 ± 29.51	39.60 ± 16.52	0.221		99.65 ± 63.33	60.87 ± 35.09	0.72
c-e rim			157.00 ± 96.14	39.60 ± 16.52	**0.009**		1 35.65 ± 83.86	60.87 ± 35.09	0.12
adjacent			91.33 ± 99.10	85.44 ± 88.08	1		31.70 ± 12.27	35.45 ± 22.76	1

*P*-values below 0.05 are boldfaced.

*Lesion* former target of SRS, *c-e rim* contrast-enhancing rim of the lesion, *Adjacent* brain parenchyma adjacent to the borders of SRS.

Summarising the results of Cho and NAA of the spectroscopic imaging, Cho was significantly higher and NAA was significantly lower than the contralateral side in the adjacent parenchyma up to the 2nd ring at the end of the observation period (Fig. 3). Cho became significantly different from the contralateral side (*P* < 0.001 for the 1^st^ ring, and *P* = 0.004 for the 2^nd^ ring, respectively) over time, even in the case of CR. NAA was significantly reduced compared to the contralateral side in the core (*P* = 0.015) at the beginning of the study period and continued to decrease. NAA became significantly lower in the 1st and 2nd ring at the end of the observation period (*P* = 0.001 for the 1st ring, and *P* = 0.043 for the 2nd ring, respectively). Lac was elevated in the 1st ring compared to the contralateral healthy brain and became evident, too, at the end of the period (*P* = 0.013). Lac levels were elevated in the core (*P* = 0.026) at the beginning of the study period and declined significantly (*P* = 0.049). In cases of CR, Lac could not be detected in any localisation. Cr in the 1st ring was initially slightly reduced and in the 2nd ring slightly increased and returned to normal values over time, which did not reach the level of significance, however.

**Fig. 4:**
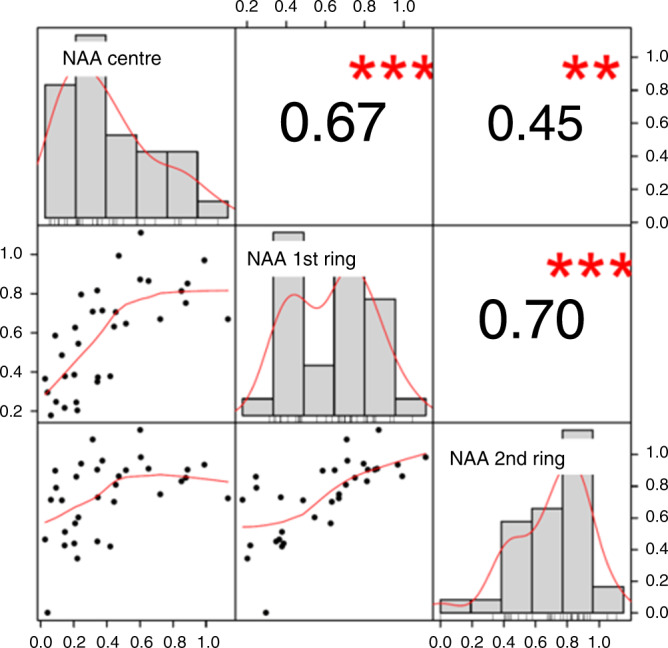
Correlation matrix for N-acetyl-aspartate (NAA) of the core, the 1st and the 2nd ring. The main diagonal displays the density function of the normalised values of NAA of each localisation. In the cells above the main diagonal, correlation coefficients and in the remaining cells the pairwise scatterplots are created.

Interestingly, NAA values correlate positively in all three locations (*r* = 0.7); for example, when the value at the contrast-enhancing part is significantly lower than at the opposite side at a certain time point, it is the same for the first and the second ring (Fig. 4). This also applies to a lesser degree to Cr (*r* = 0.35). In the case of Cho, the enhancing part behaves differently than the respective rings (r = −0.01). Cho and Cr develop in a similar way and interact positively for the core of the lesion (r = 0.49) and the second ring (r = 0.42), while NAA develops independently of all other values.

ADC of the entire lesion and of the adjacent parenchyma was constantly elevated (*P* < 0.001) (Fig. 2c) compared to the contralateral healthy brain. Interestingly, even in the four patients without residual changes on morphological MRI, the ADC was elevated at the site where the lesion was treated.

rCBV in the entire lesion (*P* < 0.001) and in the contrast-enhancing rim (*P* < 0.001) increased significantly, as did CBF in the contrast-enhancing rim (*P* = 0.009). rCBV showed a further significant increase in the entire lesion (*P* = 0.036) and the contrast-enhancing rim (*P* = 0.044) during the observation period. The perfusion parameters of the adjacent parenchyma remained normal throughout the observation time.

## Discussion

Our study shows that functional and metabolic MRI can depict radiobiological responses and their time course in the healthy parenchyma adjacent to metastases effectively treated with SRS. Our data demonstrate low values of NAA at a distance of up to 1.5 cm from the target borders of SRS associated with high values of Cho compared to the contralateral healthy side at the end of the observation period. The Lac levels in the adjacent tissue declined, whereas ADC levels remained elevated throughout the study period.

Radiation induces acute cell death and also activates molecular and biochemical cascades, which mediate a component of secondary injury and cell death.^15,16^ This response is a continuous and dynamic process. Our findings suggest that astrocytosis developing through microglial activation up to 1.5 cm from the SRS target borders is associated with permanently impaired neuronal integrity. Furthermore, we found that the injury is present despite normal-appearing conventional MRI findings and does not recover over time.

### ^1^H- magnetic resonance spectroscopic imaging

Sundgren et al.^17^ found that the NAA/Cr and Cho/Cr ratios decreased significantly in normal cerebral tissue after whole-brain radiation therapy for primary brain tumours from week 3 to 6 months. A reduction in NAA levels has also been reported to occur immediately after prophylactic whole-brain radiation therapy in patients with lung cancer.^18^ Both studies measured NAA in brain tissue that was within the field of radiation. In contrast to these studies, we noted significantly lower values of NAA than in the contralateral healthy brain not only in the target, but also out-of-field. NAA is one of the most abundant brain metabolites and is highly concentrated in neurons, representing a noninvasive marker for neuronal health, viability, and cell number. A reduced NAA is, therefore, most likely due to neuronal damage, neuronal cell death due to apoptosis, and neuronal dysfunction secondary to the irradiation.^19^ ^1^H-MRSI studies in patients with acute demyelinating lesions have shown that significant recovery of NAA can occur after acute brain damage; therefore, low values of NAA compared to the contralateral healthy brain should not always be interpreted as an index of irreversible neuronal loss.^20^ In contrast, our results suggest that SRS leads to severe long-term effects with irreversible neuronal dysfunction and neuronal loss at least up to 1.5 cm from the target.

In microscopic examinations of autopsy studies, Koike et al. could show that, after SRS, the effects of killing tumour cells were by far greater in the centre of the lesion, whereas reactive changes such as gliosis or infiltration with macrophages were more prominent towards the tumour periphery.^21^ Necrotic tissue is a convincing explanation for the positive proof of Lac in the core of the treated lesion in our data. The association of a raised Cho/Cr ratio with presence of Lac is thought to be related to oligodendrocyte damage and myelin breakdown in the presence of compromised oxidative phosphorylation, resulting in severe cerebral necrosis.^22^ Presence of Lac in ^1^H-MRSI spectra indicates carboxidative metabolism and impaired oxidative respiration and is attributed to the presence of macrophages using anaerobic glycolysis.^23^ Macrophage infiltration beyond the border of the SRS-treated target and into the adjacent tissue offers a good explanation for the elevated Lac in the adjacent brain parenchyma, the declining levels of Lac in the observation period, and the disappearance of Lac in CR observed in our study.

Microglial activation and macrophage infiltration might represent the morphological background for the additional MRS alterations that we have observed. Irradiation of the CNS activates signal transduction pathways that control cellular stress responses, which initiate inflammatory reactions^24^ and result in increased numbers of microglial cells in areas of tissue injury.^25^ Microglial activation plays an important role in phagocytosis of dead cells in the CNS and generally occurs together with reactive astrocytosis.^24,26^ Alomari et al.^27^ characterised the histopathological changes associated with enhancing versus non-enhancing regions on MRI in 14 patients treated with SRS for brain metastases. In contrast to our study, they performed serial biopsies in the follow-up of etiologically heterogeneous, progressive, gadolinium-enhancing lesions. They found coagulative necrosis in the core of the lesion and recurrent tumour growth or inflammatory demyelinating changes in the contrast-enhancing part. The elevated Cho in our study in the contrast-enhancing portion of the lesion may be explained by phagocytosis of the treated metastasis since Cho represents cell membrane precursors and also cell membrane breakdown products. Microgliosis and reactive astrocytosis generally occur together. The reactive astrocytosis in the periphery of the irradiated brain tissue reported by Alomari et al explains our observation of a continuously increasing Cho up to 180% throughout the study period at a distance of 1.5 cm from the target borders of SRS. Cho is known to be related to cell density, cell proliferation, and macrophage infiltration.^28^

These results might have clinical impact since such elevated Cho/Cr ratios have been reported in the peritumoural region of gliomas but not in metastases.^29^ In the follow-up of both entities it has been suggested that radiation-induced necrosis could be distinguished from tumour recurrence, which is characterised by a high Cho/NAA ratio.^22,30^ However, high levels of Cho were recently reported in some cases of radiation necrosis initially suspected of being brain tumour recurrence.^31^ Our results show, firstly, that Cho values are clearly elevated in the tissue adjacent to the irradiated metastasis even in the case of CR. This important finding should be considered when differentiating radiation necrosis from tumour recurrence by MRS. Secondly, there is a temporal dependency, as Cho increased during the study period.

Interestingly, Cr in the second ring returned to normal values at the end of the study period after an initial, though not significant, increase up to 125%. Cr plays a vital role in the storage and transport of cellular energy through the Cr kinase reaction generating adenosine triphosphate.^32^ As Cho showed increasing values as a result of gliosis and NAA at the same time was decreased owing to the decrease in neuronal cell population at a distance of 1.5 cm from the target borders of SRS, the normalisation of Cr in the second ring could be a result of a no longer increased requirement of phosphate stores by Cr over time.

### Diffusion-weighted magnetic resonance imaging

Autopsy reports assessing the effectiveness of SRS have mainly focused on brain metastasis in terms of residual tumour volume.^21,33^ Single autopsy cases confirmed tumour necrosis free from active tumour cells in the core area of the brain metastases 14–17 days^33^ and 21 days after SRS.^21^ These findings are in line with the elevated ADC values in the core of the SRS target in our study. DWI can assess tissue microstructure and their pathological processes by characterising the microscopic diffusion of free water molecules, the magnitude of which is quantified as ADC.^34^ While restricted diffusion with low ADC values is a consequence of cytotoxic oedema or increased cellularity, increased diffusion of water molecules occurs in tissues with either decreased cellularity, such as necrotic tissues or necrotic tumours or in vasogenic oedema.^35^ The latter offers a reasonable explanation for the continuously elevated ADC values we observed in both the core of the SRS target and the adjacent healthy brain.

### Dynamic susceptibility-weighted contrast-enhanced magnetic resonance imaging

DSC MRI of the brain provides haemodynamic information about relative blood volume and flow, characterising the microvascular environment in the tissue. The hypothesis of a continued deterioration of vascular function as the primary radiation-induced factor has been widely accepted.^22^ Perfusion measurements could be a valuable aid in monitoring treatment to distinguish between recurrent tumour and treatment-related changes such as radiation necrosis. In a prospective study of 20 patients, Sugahara et al.^36^ found that a normalised rCBV greater than 2.6 was indicative of tumour recurrence, whereas a value below 0.6 was indicative of radiation necrosis. In this regard our results might have clinical impact. While the perfusion parameters rCBV and rCBF were continuously elevated in the target region, they were normal in the adjacent tissue. The results of our study suggest that continuous repair and removal as part of the healing processes of the SRS-treated metastasis can increase perfusion parameters.

### Limitations

There are limitations that should be considered in the current study. First, the number of included lesions is relatively small. However, we studied these lesions with 41 multiparametric MR examinations over a long observation period of up to 142 weeks, giving insight into pathological processes over time. Secondly, we did not obtain histopathological confirmation. However, as mentioned above, a biopsy to prove response to treatment would not have been ethically acceptable. Thirdly, the included patients had a heterogeneous group of primary tumours. However, metastases from radioresistant tumours, such as melanoma and renal cell carcinoma, respond to SRS as do metastases from radiosensitive tumours.^2,37^ Finally, we did not compare our results with other modalities, such as PET-CT. Including a larger number of patients, correlating the functional and metabolic MRI results with individual neuropsychological changes, and validating against other modalities would increase confidence in the conclusions of this study. Thus, further comparative studies are warranted.

### Conclusions and future directions

Functional and metabolic MRI can characterise radiobiological responses and their time course in the healthy brain parenchyma adjacent to metastases effectively treated with SRS. Our data underline the hypothesis that the long-term response is a continuous and interacting process. Our study provides evidence of reduced NAA up to 1.5 cm from the target borders of SRS associated with elevated Cho at the end of the observation period. The Lac levels declined in the adjacent tissue, whereas ADC levels remained elevated. Enhancing our understanding of these responses could lead to dedicated strategies for developing neuroprotective drugs targeted at different steps of radiation-induced disease mechanisms. To mitigate the cytotoxic effects of radiation in normal tissue, drug treatments, such as antioxidants and antiapoptotic and anti-inflammatory agents and growth factors are subjects of research.^38^ Combined functional and metabolic MR techniques may be suited for monitoring the timely stratification of patients for these treatments.

## Data Availability

The authors declare that all data supporting the findings of this study are available within the article or are available from the corresponding author upon reasonable request.
